# Towards standardised cross-sectoral agreements for data depositing and data access in Trusted Research Environments: streamlined contracting to advance public benefit research.

**DOI:** 10.23889/ijpds.v9i5.2626

**Published:** 2024-09-18

**Authors:** Rachel Brophy, Cassie Smith, Andy Boyd, Steering Group Pan UK Data Governance

**Affiliations:** 1 HDR UK; 2 University of Bristol; 3 UK Health Data Research Alliance

## Objective

Trusted Research Environments (TREs) rely on Data Contributor Agreements (DCAs) and Data Access Agreements (DAAs) to bind organisations to terms and conditions in place to protect data. Our objective is to develop and drive adoption of DCA and DAA standardised templates which are optimised for data science in TREs. This is intended to reduce delays arising from the legal review process yet must not compromise on the safeguards needed for trustworthy data science.

## Approach

A template DAA and DCA have been developed by the Pan-UK Data Governance Steering Group of the UK Health Data Research Alliance, underpinned by a core set of principles, and informed by a benchmarking exercise of existing DCAs/DAAs and meaningful involvement of public members and stakeholders.

## Results

Principles for TRE data contribution and data access in TREs have been mapped to core DCA and DAA terms for UK-based TREs and assigned to Five Safes’ categories. Flexibility will be ensured by including terms specific to TREs or specific data/data owners in customisable annexes to the agreements. Public views obtained through public involvement and engagement (PIE) activities have been formative in the development of the outputs.

## Conclusions

By providing a familiar structure and terms, the templates should build trust among data owners and the UK public and provide clarity to all parties on their data protection obligations.

## Implications

This familiar structure and terms are intended to accelerate   data research by enabling faster ingress of data into TREs and approval of projects, enabling more timely research.

